# The effect of internal, external, and holistic focus of attention on standing long jump performance in novice and skilled karatekas

**DOI:** 10.1002/ejsc.12152

**Published:** 2024-06-12

**Authors:** Tooran Noroozi, Esmaeel Saemi, Mohammadreza Doustan, Harjiv Singh, Christopher A. Aiken

**Affiliations:** ^1^ Department of Motor Behavior and Sport Psychology Faculty of Sport Sciences Shahid Chamran University of Ahvaz Ahvaz Iran; ^2^ Department of Kinesiology and Nutrition Sciences University of Nevada Las Vegas Nevada USA; ^3^ Orlando Magic Basketball Club Orlando Florida USA; ^4^ Department of Kinesiology New Mexico State University Las Cruces New Mexico USA

**Keywords:** attentional focus instructions, karate, motor performance, skill level

## Abstract

An important application for training instructions is found in directing one's attentional focus. This direction can occur in different internal, external, or holistic forms. However, comparison between these three forms of instructions is a relatively recent development rarely reported at different skill levels or various sports including karate. Therefore, the present study attempts to investigate the effects of three forms of instructions on standing long jump performance in skilled and novice karatekas. The participants were 60 skilled and novice karatekas (all females; mean age: 21.32 ± 1.65) who completed 12 standing long jump trials under different focus conditions (3 trials for each condition: internal, external, holistic and control condition) in a counterbalanced order. Our findings suggested significant main effects, indicating that skilled karatekas outperformed the novices. The findings also showed that regardless of skill levels, the participants exhibited similar performance under external and holistic focus conditions while performance in both of these conditions was superior compared to performance under internal focus and control conditions. No difference was observed between the control and internal focus conditions. It seems that skilled and novice karatekas may benefit from holistic and external focus of attention instructions which enhance their motor performance. Thus, it is recommended that coaches should incorporate these two types of attentional focus instructions into their training sessions.

## Highlights

- Both external as well as holistic focus of attention are similar and improve standing long jump performance.
- Both skilled and novice karatekas benefit from external as well as holistic focus of attention.
- Performance under an Internal focus of attention is similar to control conditions and does not benefit standing long jump performance.

## 1 INTRODUCTION

Martial arts are among the sports which have received considerable interest in motor learning and biomechanics research studies (Machado et al., 2010; Mattes, 2016). One of the most popular martial arts across the world is karate, where motor skills need to be performed using great force, speed, and power (Cronin & Sleivert, 2005; Koropanovski et al., 2011). While it is important to assess muscular strength and perceptual motor ability in karate, it is of particular importance to assess jumping ability as there is a positive relationship between increased jumping performance and scoring in karate athletes (Sant'ana et al., 2014; Yildiz, 2018). Therefore, utilizing motor behavior research to improve jumping performance can help karate athletes achieve success in this sport (Ravier et al., 2004).

Scientific evidence shows that attention, and how it is directed during performance of sports skills, can significantly improve or degrade motor performance and learning (for a review see Chua et al., 2021). Attentional focus is commonly manipulated by internal, external, and more recently, holistic foci. An internal focus is when an individual focuses on body movements during task performance while an external focus is when an individual focuses on the intended effects of the movements on the environment (Wulf, 2013). Furthermore, instead of specific movement details, if the individual focuses on the general sense or feeling of the movement that individual can be said to have adopted a holistic focus of attention (Becker et al., 2019; Saemi et al., 2023).

In recent years, many studies have sought to identify the most effective type of attentional focus and how it may influence motor performance (Abdollahipour et al., 2017; Asadi et al., 2019; Coker, 2016, 2018; Lohse et al., 2010, 2014; Porter et al., 2010, 2012, 2013; Saemi et al., 2017, 2023; for a review see Chua et al., 2021). The findings of these studies mostly highlight that an external focus of attention (i.e., focus on the intended effect of a movement in the environment) effectively improves motor learning and performance compared to internal focus of attention (i.e., focus on body movements) (for a review see Chua et al., 2021). Wulf et al. (2001) put forth the constrained action hypothesis to explain the benefits on an external focus by suggesting that consciously controlling an action with an internal focus will constrain the motor system and inhibit automatic processes that control movement. In contrast, shifting the attention away from a movement and focusing on the effects of that movement (i.e., external focus of attention) will enable self‐organization in the motor system through automatic processing (McNevin et al., 2003; Wulf et al., 2001). The predictions based on this hypothesis has been so far supported by kinematics, kinetics, and performance data (Asadi et al., 2019; Coker, 2018; Lohse et al., 2010; Vance et al., 2004).

The benefits of an external focus compared to an internal focus has been reported in various jumping tasks (Asadi et al., 2019; Becker et al., 2019; Coker, 2016, 2018; Wulf et al., 2007; for a review see Makaruk et al., 2020). These findings have been observed for both skilled and novice performers. Asadi et al. (2019) found that both skilled as well as novice participants jumped farther under an external focus (try to focus on the cone that is in front of you) compared to an internal focus (try to focus on extending your knees). Additional studies have also shown the benefit of an external focus to increase jumping performance in both skilled and novice performers (Porter et al., 2010, 2012, 2013). However, some other studies have also reported conflicting results in similar jumping tasks (Ericksen et al., 2024; Moran et al., 2024). For example, Moran et al. (2024) did not report a significant difference between internal and external foci in a jumping task for elite academic soccer players. Therefore, the need for more research on jumping task with considering different skill levels is still felt.

Alternatively, recent studies have discussed another kind of attentional focus instruction known as holistic focus (i.e., focus on the overall feeling of movement) (Abedanzadeh et al., 2022; Becker et al., 2019; Saemi et al., 2023; Shooli et al., 2024; Zhuravleva & Aiken, 2023; Zhuravleva et al., 2023), which may be used as an alternative focus to avoid an internal focus of attention when an external focus is not appropriate. There have been a few frameworks forwarded to explain the benefits for a holistic focus of attention. Zhuravleva et al. (2023) argue that a holistic focus allows a performer to direct attention to skill‐relevant sensations, while avoiding an internal focus toward body correction. The action effect principle of the common coding theory (Prinz, 1990) suggests that actions are planned based on the desired outcome of the movement. A holistic focus provides an alternative to an external focus by still providing a task relevant cue that directs attention to a desired outcome of the movement (e.g., smooth or explosive). Furthermore, Wulf and Prinz (2001) suggest that some skills require a focus instruction that is directed toward a close effect or more of a kinesthetic effect, while others require a remote effect, or effect on the environment. An important aspect of a holistic focus is that it can provide relatable cues to performers in situations where an external focus is not appropriate. Directing attention away from an internal focus seems to be a key to improving performance.

Becker et al. (2019), in the first study on holistic focus of attention, demonstrated that college students jumped significantly farther while adopting a holistic focus (‘focus on making your movement feel explosive’) and an external focus (‘focus on jumping as close to the orange cone as possible’) compared to an internal focus (‘focus on extending your knees’) while performing a standing long jump. Moreover, Abedanzadeh et al. (2022) expanded these findings by showing that both a holistic and an external focus of attention facilitated motor learning of a badminton serve compared to an internal focus. However, Saemi et al. (2023) found that the effectiveness of attentional focus instructions in their different forms is influenced by athletes' skill levels. They showed that experienced children swimmers exhibited better performance under external focus conditions compared to internal and control conditions, while no difference was found under holistic conditions. As far as novice children swimmers were concerned, they did not find any difference between control, internal, external, and holistic focus instructions. Furthermore, Becker et al. (2020) showed that external focus of attention was superior to internal and holistic focus for sample entropy in a balance task. More recently, Zhuravleva and Aiken (2023) compared different forms of attentional focus on motor performance in a shot put task. Eighteen skilled female track and field athletes completed the task under three conditions: external, internal, and holistic focus of attention. It was observed that athletes threw the shot put significantly further under a holistic focus compared to internal focus. No significant difference was found between internal and external conditions, or between external and holistic conditions.

Similarly, Zhuravleva et al. (2023) examined standing long jump performance in skilled female track and field athletes utilizing various attentional focus strategies. They found that athletes jumped significantly further under holistic focus conditions compared to internal focus conditions. No significant differences were found between holistic and external or external and internal. One limitation demonstrated by Zhuravleva et al. (2023) and Saemi et al. (2023) is the absence of a control condition along with the other three focus of attention conditions. Moreover, given the emphasis on larger sample sizes to reduce type I error in behavioral research studies (Lohse et al., 2010) and the small sample size used by the recent research studies in this area (Asadi et al., 2019; Zhuravleva et al., 2023), it seems highly necessary to conduct further research in this area with larger sample sizes.

Given that skill level in different sports is likely to have impact on how different attentional focus instructions influence outcomes (Saemi et al., 2023), further research is still needed in this area within a variety of sports and activities. With the popularity around the world for karate, it is important to understand how various attentional focus strategies influence karatekas in performance and training. The use of a holistic focus may be of importance as previous high‐level coaches report the use of holistic cues in their coaching and instruction (Zhuravleva et al., 2023). The present study seeks an answer to the question of whether using internal, external, holistic, and no instructions (control) influence performance measures like standing long jump performance among karatekas, and whether these instructions influence skilled and novice karatekas differently.

## 2 METHOD

### 2.1 Participants

The participants were 60 female karatekas (mean age: 21.32 ± 1.65) at skilled and novice levels who were selected through convenient sampling. The sample size was evaluated using G*Power 3.1. Given the values of *α* = 0.05, 1 − *β* = 0.95, effect size = 0.25 (Zhuravleva et al., 2023), and 2 (skill levels) × 4 (attentional focus conditions) mixed analysis of variance (ANOVA) with repeated measure, a sample size of 36 was reported. However, due to potential attrition and the importance of increased sample size highlighted by behavioral studies (Lohse et al., 2016), a total of 60 subjects at two skill levels, that is, skilled (*N* = 30) and novice (*N* = 30), participated in the study. We included individuals who (1) were self‐reported as being physically healthy and did not have any muscle or joint injury and (2) were between 20 and 24 years of age. The criteria for selecting participants based on skill level was as follows: skilled karate participants were individuals who had (1) at least 5 years of karate experience, (2) at least a brown belt in karate, and (3) individuals who were competing in the country's karate 1‐premier league at the time of the study. On the other hand, the novice karate participants were individuals who (1) had a maximum of 5 months of karate experience and (2) had a yellow belt in karate. However, we excluded individuals who were not willing to participate in the study. All participants completed the informed consent form before taking part in the study. The study protocol received approval from the local institutional review board and complied with the Declaration of Helsinki.

### 2.2 Apparatus and instruments

The standing long jump task was performed on a 1.5 m × 4.5 m rubberized mat to the measure jump distance. A white strip was taped on the flooring to mark the starting line where the participants stood with their toes behind the line prior to each trial. Participants were instructed to jump off two feet, the distance from the starting line to the back of the heel closest to the starting line was recorded in centimeters as the participant's jump distance. A cone was located at a distance of 5 m from the participants. This procedure has been used to measure standing long jump performance consistently in the motor behavior literature (Asadi et al., 2019; Becker et al., 2019).

### 2.3 Procedure

Participants were asked to perform a standing long jump. For this task, three trials were performed under each of four focus conditions (internal, external, holistic, control) for a total of 12 trials in a counterbalanced order. All attentional focus instructions were developed based on the previous studies (Asadi et al., 2019; Becker et al., 2019). Prior to starting the experimental protocol, participants were briefed on the experiment and were provided informed consent. Participants then completed a 5‐min warm‐up phase which consisted of stretching movements and slow running. Next, they rested for 1 minute, during which they received instructions about the correct placement of their feet to perform the standing long jump. All participants were instructed to jump as far as possible during all trials. Under the internal focus conditions, participants were instructed to “mentally focus on extending your knees during the jump”. For the external focus conditions, the participants were told to “mentally focus on the cone placed in front of you.” In the holistic focus condition, participants were told to “mentally focus on the fluidity of your movement.” No additional attentional instructions were provided under the control condition. These instructions were based on previous literature (Asadi et al., 2019; Becker et al., 2019). After each experimental condition (i.e., internal, external, and holistic), the participants answered a focus of attention compliance questions. A visual analog scale that ranged from 0 to 100 was used to assess compliance for each question. To examine compliance for an internal focus, participants were asked to rate to what extent they focused on, “While executing the skill, I was focused on extending my knees during the jump”. For an external focus participants were asked, “While executing the skill, I was focused on the cone placed in front of mine”, and for the holistic focus condition, participants were asked, “While executing the skill, I was focused on the fluidity of my movement”.

### 2.4 Data analysis

For data analysis, a one‐way ANOVA was used to compare the demographic characteristics between the skilled and novice groups. A 2 (skill levels) × 4 (focus conditions) mixed factors ANOVA with repeated measures was used to analyze the jump distances. To compare the compliance scores for control and each experimental condition, four 2 (skill levels) × 3 (focus conditions) mixed factors ANOVAs were conducted with repeated measure on the last factor. Bonferroni post‐hoc test was applied to assess any significance between conditions. The data were analyzed in SPSS 26 with an alpha level of 0.05.

## 3 RESULTS

Our initial findings indicated that the data were normally distributed (Shapiro‐Wilk test was not significant for all data, *p* > 0.05). No demographic difference for age, height, and weight were observed between the skilled and novice groups. A significant difference between the two groups was found only in sports history (Table 1).

**TABLE 1:** Participant's demographic information.

Demographic characteristics	Groups (M ± SD)		Significance level
	Skilled	Novice	
*N*	30	30	‐
Age (year)	21.60 ± 1.75	21.03 ± 1.52	0.18
Height (cm)	162.73 ± 6.69	160.33 ± 18.18	0.28
Weight (kg)	58.83 ± 9.18	63.70 ± 23.13	0.50
Sports history (year)	10.19 ± 3.18	0.87 ± 0.31	0.0001^a^

^a^ Significant at *p* ≤ 0.05.

### 3.1 Standing long jump performance

**FIGURE 1:**
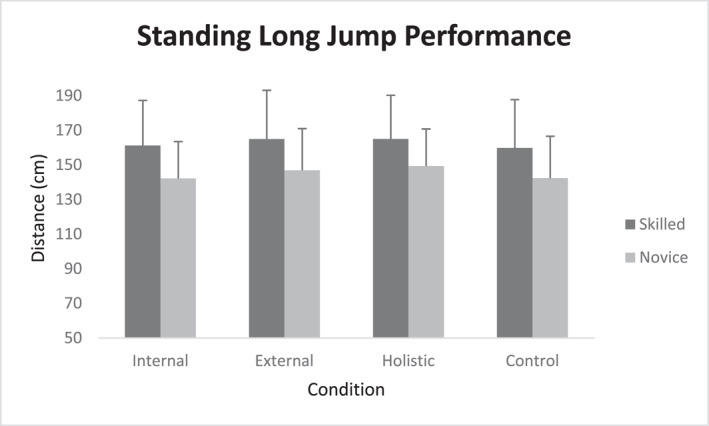
The standing long jump performance for all attentional conditions as well as two skill levels. Error bars represent standard deviation.

The 2 (skill levels) × 4 (focus conditions) mixed factors ANOVA with repeated measure was not normally distributed. Greenhouse–Geisser corrections were applied and reported. The findings indicated a significant main effect for skill levels (*F* (58, 1) = 7.91, *p* = 0.007, partial *η* ^2^ = 0.12) and focus conditions (*F* (2.31, 134.14) = 9.96, *p* = 0.0001, partial *η* ^2^ = 0.14). However, the interaction of skill levels and focus conditions was not reported to be significant (*p* = 0.61). As the main effect for group was significant, it was found that skilled karatekas (162.74 ± 4.41) outperformed novice performers (145.18 ± 4.41, *p* = 0.007) in the standing long jump task, regardless of the attentional focus instruction. The results from Bonferroni post‐hoc test on pairwise comparison of focus conditions indicated that both external (155.91 ± 3.38) and holistic focus (157.18 ± 3.02) were superior to internal focus (151.65 ± 3.11; *p* = 0.02 and 0.001, respectively) and control conditions (151.10 ± 3.37; *p* = 0.001 and 0.002, respectively). In other words, both novice and skilled athletes exhibited better standing long jump performance under external and holistic focus conditions compared to control and internal focus conditions. No difference was observed between the control and internal focus conditions (Figure 1).

### 3.2 Compliance check

**FIGURE 2:**
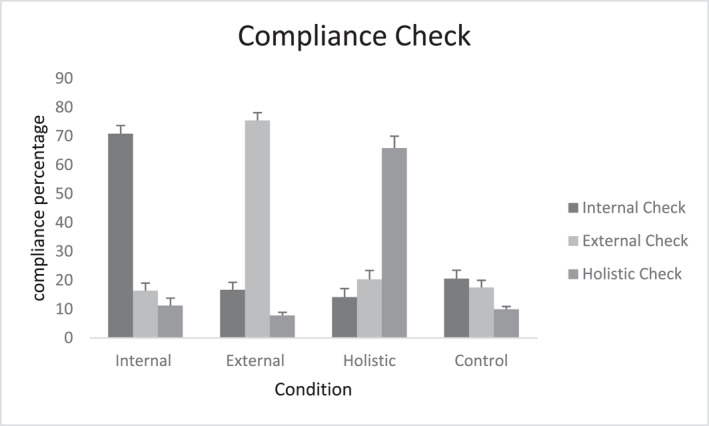
The mean and SD of the compliance check questions for all attentional conditions. Error bars represent standard deviation. Internal Check: the degree of the compliance for an internal focus, External Check: the degree of the compliance for an external focus, Holistic Check: the degree of the compliance for a holistic focus.

Under the internal focus conditions, the initial findings supported Mauchly's sphericity assumption (*p* > 0.05). Therefore, the original values were reported. The findings indicated that only the main effect for focus condition was significant (*F* (2, 16) = 102.16, *p* = 0.0001, partial *η* ^2^ = 0.63). Other main effects for skill levels and interaction were not reported to be significant (*p* = 0.29 and *p* = 0.50, respectively). The results from Bonferroni post‐hoc test, regardless of skill levels, showed significant difference between internal focus condition (70.83 ± 2.82, *p* = 0.001) and external (16.41 ± 2.61) and holistic (11.25 ± 2.59, *p* = 0.001) conditions. No difference was found between other conditions. In other words, the participants under internal focus conditions were focused on the internal focus instruction (Figure 2).

Under external focus conditions, the initial findings rejected Mauchly's sphericity assumption. Thus, Greenhouse–Geisser corrections were reported. The findings showed that only the main effect for focus conditions was significant (*F* (1.66, 96.57) = 154.20, *p* = 0.0001, partial *η* ^2^ = 0.72). Other main effects for skill level and interaction were not reported to be significant (*p* = 0.16 and *p* = 0.67, respectively). The results from Bonferroni post‐hoc test, regardless of skill levels, showed that external focus conditions (75.41 ± 2.73) were significantly different from internal focus condition (16.66 ± 2.66, *p* = 0.001) and holistic focus condition (7.81 ± 1.08, *p* = 0.001). A significant difference was also reported between internal focus condition and holistic focus condition (*p* = 0.033). In other words, the participants under external focus conditions notably focused on external focus instruction (Figure 2).

Under holistic focus condition, the initial findings rejected Mauchly's sphericity assumption (*p* < 0.05). Thus, Greenhouse–Geisser corrections were reported. The findings showed that only the main effect for focus condition was significant (*F* (1.59, 92.71) = 45.91, *p* = 0.0001, partial *η* ^2^ = 0.44). Other main effects for skill levels and interaction were not reported to be significant (*p* = 0.31 and *p* = 0.24, respectively). The results from Bonferroni post‐hoc test, regardless of skill levels, showed that holistic focus conditions (65.83 ± 4.16) were significantly different from external focus condition (20.33 ± 3.07, *p* = 0.001) and internal focus condition (14.16 ± 2.96, *p* = 0.001). No significant difference was found between other conditions. In other words, the participants under holistic focus condition considerably focused on holistic cues (Figure 2).

Under control condition, the initial findings confirmed Mauchly's sphericity assumption (*p* > 0.05). Therefore, the original values were reported. The findings indicated no significant main effect for focus conditions, skill levels, and interaction (*p* = 0.06, *p* = 0.95, and *p* = 0.21, respectively). In other words, under control condition, the participants did not have any particular focus of attention (Figure 2).

## 4 DISCUSSION

The present study aimed to compare effects of different attentional focus instructions under internal, external, holistic, and control conditions on standing long jump performance of skilled and novice karatekas. Our findings indicated that skilled karatekas significantly outperformed novice ones on jump distance for the standing long jump. The findings also showed that, regardless of skill levels, jumping under external and holistic focus conditions improved motor performance compared to internal focus and control conditions.

These results are consistent with a considerable amount of attentional focus literature where an external focus was significantly better for performance than an internal focus for both skilled (e.g., Ille et al., 2013; Porter et al., 2012; Saemi et al., 2017, 2023; Shooli et al., 2024; Wulf & Su, 2007; Zhuravleva & Aiken, 2023) and novice performers (e.g., Abdollahipour et al., 2017; Abedanzadeh et al., 2022; Asadi et al., 2022; Lohse et al., 2014; Palmer et al., 2017). The non‐significant effect between the focus of attention and skill levels is not new, and to be expected. In a review by Chua et al. (2021), it is highlighted that adopting an external focus of attention benefits performance regardless of skill levels. Similarly, a benefit to a holistic focus of attention has been observed in skilled performers (Shooli et al., 2024; Zhuravleva & Aiken, 2023; Zhuravleva et al., 2023) and novice performers (Abedanzadeh et al., 2022; Becker et al., 2019). One paper that was in contrast to our reported findings was that of Saemi et al. (2023) who observed that only experienced children swimmers, and not novice children, performed better with an external focus of attention compared to internal focus of attention. One potential reason for the contrast in this study is the difference in ages of participants. Skill levels could also be different with the differences in ages between participants (Saemi et al., 2023).

One theoretical approach that explains the benefit of an external focus over internal focus is the constrained action hypothesis (Wulf et al., 2001). According to the constrained action hypothesis (Wulf et al., 2001), the adoption of an internal focus of attention can hinder motor learning and performance due to interference with automatic control. The adoption of an external focus of attention can improve motor learning and performance due to the facilitation of automatic motor control and self‐organized actions. Therefore, both novices and skilled groups in the present study seem to have been able to improve their standing long jump performance as a result of facilitated automatic process and self‐organized actions under external focus condition, while under internal focus condition they exhibited a performance drop due to inhibited automatic processes and performance at a conscious control level. Furthermore, some studies have shown that the superiority of the external focus of attention, and especially the distal external focus of attention, can be due to the improvement of functional movement variability during the adoption of external attention compared to internal attention (Singh et al., 2022). In other words, adopting an external focus can lead to optimizing compensatory coordination of body parts during skill execution (Singh et al., 2022). It is probable that in this study, participants in the external focus condition experienced optimal compensatory coordination between their body parts, which ultimately led to the improvement of their jumping performance. However, this requires future investigation. In addition, based on other related theories, such as the OPTIMAL theory of motor learning (Wulf & Lewthwaite, 2016), adopting an external focus of attention over an internal focus of attention can provide a more efficient coupling between action and goal. Therefore, it seems that one of the reasons for the superiority of the participants in the current research study in the external focus condition was the better goal–action coupling in the standing long jump task.

The evidence in support of an external focus is still met with contradictory results. Some studies have not reported improved performance under an external focus compared to an internal focus, which is in contrast to the constrained action hypothesis (Wulf et al., 2001). For example, in a study on novice swimmers, Saemi et al. (2023) found no benefits to swimming performance from adopting an external focus of attention. In addition, Aiken and Becker (2023) showed that advantages of internal or external focus of attention for learners depends on when focus of attention is presented. For example, disadvantages of an internal focus were only observed during performance of the skill, not during movement preparation.

Our findings also indicate that adopting a holistic focus is as beneficial as adopting an external focus in standing long jump performance of novice and skilled karatekas. These findings are in line with current research studies in this area, although the number of studies are relatively limited (Abedanzadeh et al., 2022; Becker et al., 2019; Shooli et al., 2024; Zhuravleva & Aiken, 2023; Zhuravleva et al., 2023). For instance, Becker et al. (2019) showed that when performing a standing long jump, novice performers exhibit a better performance under external and holistic focus conditions than under an internal focus condition. These findings are in line with our findings on novice karateka which found that external and holistic focus conditions similarly resulted in better performance compared to internal focus and control conditions. It is plausible that a holistic focus facilitates automatic processes in the motor system similarly to an external focus (Wulf et al., 2001). This is particularly impactful as many motor skills do not easily lend themselves to an external focus instruction that makes sense to the performers. Zhuravleva and Aiken (2023) showed that performing under a holistic focus condition can enhance performance for an underhand shot put toss for skilled athletes, which is in line with the findings from the current study. Similar findings where a holistic focus significantly improved performance compared to an internal focus has been reported in a standing long jump with college track and field athletes (Zhuravleva & Aiken, 2023), as well as basketball free‐throws with skilled basketball players (Shooli et al., 2024). In general, our findings together with previous studies suggest that the benefit of a holistic focus over an internal focus can be generalized to many motor skills (Abedanzadeh et al., 2022; Becker et al., 2019; Shooli et al., 2024; Zhuravleva & Aiken, 2023; Zhuravleva et al., 2023). Therefore, it seems that coaches in different sports environment, particularly for novice and skilled karatekas, can use holistic or external focus of attention instruction to facilitate improvements in athletes' motor performance.

As one of the earliest studies of its kind to examine the effects of internal, external, and holistic focus instructions on standing long jump performance of skilled athletes, Zhuravleva and Aiken (2023) showed that holistic focus outperforms internal focus and can improve motor performance in skilled individuals. However, further investigation was needed due to the absence of a control condition where participants received no attentional instructions. Our study can provide coaches with more information than that provided by previous studies since it included a control condition and demonstrated a benefit for both external focus and holistic focus conditions over internal focus and control conditions. In addition, following Saemi et al. (2023) who investigated the effects of internal, external, and holistic focus conditions on swimmers at skilled and novice levels, our study is the second one of its kind that simultaneously examined these three (internal, external, and holistic) conditions and compared them to a control condition for karatekas at skilled and novice levels. Another advantage of our study stems from its sample size. For example, as similar previous studies in this domain, Saemi et al. (2023) recruited 14 participants at any skill level, Zhuravleva et al. (2023) examined 16 skilled participants, and Zhuravleva and Aiken (2023) had 18 participants at collegiate sports level. Given the emphasis put on larger sample sizes in behavioral studies (Lohse et al., 2016), it seems that the findings of our study (with 60 participants, 30 at each skill level) can be used more confidently by coaches and athletes, particularly in karate.

Like other studies, our study had a number of limitations. For example, we only assessed standing long jump performance of karatekas. Future studies are recommended to examine other specialized performances including kicks and hand strikes for novice and skilled players under different attention focus conditions in order to provide a better understanding of applications of attentional instructions in this sport. We only studied immediate performance, future studies can consider motor learning and examine the effects of different internal, external, and holistic focus instructions on motor learning for karate skills.

Furthermore, the results from compliance check indicated that in each condition (i.e., internal, external, and holistic focus) the participants used the instructions developed for that condition. Therefore, the effects of each condition can be more confidently linked to the instructions received under each condition. Our findings also suggested that under the control condition, neither the novice nor the skilled group utilized any particular attentional focus. Based on our findings and those found by Porter et al. (2010), it is recommended that coaches should integrate the manipulation of attentional focus strategies, and particularly external or holistic focus instructions, into their training programs. Application of these attentional instructions seems to facilitate motor performance in athletes, particularly in karatekas.

## CONFLICT OF INTEREST STATEMENT

The authors declare that the study was conducted in the absence of any commercial or financial relations that could be construed as a potential source for conflict of interest.
